# Machine Learning Approach to Characterize Ferromagnetic La_0.7_Sr_0.3_MnO_3_ Thin Films via Featurization of Surface Morphology

**DOI:** 10.1002/advs.202417811

**Published:** 2025-04-26

**Authors:** Sanghyeok Ryou, Jihyun Lim, Minwoo Jang, Kitae Eom, Sunwoo Lee, Hyungwoo Lee

**Affiliations:** ^1^ Department of Physics and Department of Energy Systems Research Ajou University Suwon 16499 Republic of Korea; ^2^ Department of Computer Engineering Inha University Incheon 22212 Republic of Korea; ^3^ Department of Electronic Engineering Gachon University Seongnam 13120 Republic of Korea

**Keywords:** classifations, ferromagnetic, La_0.7_Sr_0.3_MnO_3_, machine learning, surface morphology

## Abstract

Ferromagnetic perovskite oxides, particularly La_0.7​_Sr_0.3_MnO_3_ (LSMO), show significant promise for spintronics and electromagnetic applications due to their unique half‐metallicity and colossal magnetoresistance properties. These properties are known to arise from Mn‐O‐Mn double‐exchange interactions, which are directly related to microscopic lattice structures. However, since the microscopic structure in LSMO is highly sensitive to various material parameters, such as thickness, lattice strain, oxygen deficiency, and cation stoichiometry, understanding the intricate relationship between the microscopic structures and the resulting physical properties of LSMO remains challenging. Herein, a machine learning approach is introduced to characterize ferromagnetic LSMO thin films by featurization of their surface morphology. Using an ensemble machine learning method, the non‐linear correlations between surface morphology and the electronic, magnetic properties of LSMO thin films are captured and modeled. Based on these estimated correlations, LSMO thin films are classified into five representative types, each characterized by distinctive properties and surface morphologies. These results imply that surface morphology can reveal hidden information about the strongly correlated properties of ferromagnetic LSMO thin films. Consequently, the machine learning‐based approach provides an efficient method for understanding the correlated material properties of ferromagnetic oxides and related materials through surface morphology analysis.

## Graphical abstract

A machine‐learning approach is presented to characterize ferromagnetic LSMO thin films by featurizing their surface morphology. Through an ensemble model, the non‐linear correlations between surface morphology and the electric/magnetic properties of LSMO thin films are successfully captured. This result demonstrates that surface morphology can effectively reveal essential information about the correlated physical properties of ferromagnetic oxide materials.

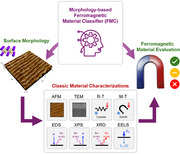

## 1 Introduction

Ferromagnetic perovskite oxides are highly attractive materials with potential applications in spintronics and magnetoelectric applications.^[^ ^1^ ^]^ La_0.7_Sr_0.3_MnO_3_ (LSMO) is a representative ferromagnetic perovskite oxide, which has drawn significant attention due to its half‐metallicity and magnetoresistance (MR) properties.^[^ ^2^ ^]^ In particular, the colossal magnetoresistance (CMR) of LSMO,^[^ ^3^, ^4^ ^]^ an extremely large MR in a temperature range near the ferromagnetic ordering of manganese spins, has shown promising potential for applications in magnetic‐field sensors, bolometers, magnetic tunnel junctions, and magnetoelectric memories.^[^ ^5^, ^6^, ^7^, ^8^, ^9^, ^10^, ^11^, ^12^ ^]^ In addition to the excellent electronic and magnetic properties, LSMO is compatible with many lattice‐matched oxides, enabling the epitaxial growth of single‐crystalline thin films and heterostructures. Previous studies have demonstrated that, importantly, physical properties of the epitaxially‐grown LSMO thin films are sensitively dependent on various material parameters, such as thickness,^[^ ^13^ ^]^ lattice strain,^[^ ^14^, ^15^, ^16^ ^]^ cation stoichiometry,^[^ ^17^, ^18^ ^]^ and oxygen deficiency,^[^ ^19^, ^20^ ^]^ which are significantly affected by the synthesis and characterization processes.^[^ ^21^, ^22^ ^]^ These material parameters directly or indirectly influence the Mn‐O‐Mn double‐exchange interactions, resulting in different electronic and magnetic properties. The relationships between these material parameters and the resultant physical properties have often been explained by the microscopic changes in crystallographic structure, such as the distortion of MnO_6_ octahedron^[^ ^23^ ^]^ and Mn‐O‐Mn bond angles,^[^ ^24^ ^]^ compared to bulk LSMO. Thus, understanding the effect of various material parameters on the microscopic structure of LSMO is crucial for optimizing and reliably reproducing its physical properties. However, precisely assessing subtle structural changes and thereby identifying correlations between the material parameters and LSMO properties present significant challenges. Furthermore, the correlations between the microscopic structures of LSMO and various material parameters are known to be intricately intertwined,^[^ ^13^, ^18^ ^]^ with each influencing the other, making a comprehensive understanding difficult.

Surface morphology may offer an efficient way to characterize the ferromagnetic LSMO thin films. The surface structure of epitaxially‐grown LSMO films is known to be governed by several synthesis conditions, such as temperature, oxygen partial pressure, and surface state of substrates.^[^ ^17^, ^19^ ^]^ These condition parameters directly affect the surface diffusivity of LSMO adatoms on the substrate, altering the growth mode and surface morphology. The surface structure is also influenced by the stoichiometry of LSMO. When excess cation atoms are present, they often cluster on the surface, forming characteristic granular morphology.^[^ ^20^ ^]^ It is important to highlight that these condition parameters, which determine the surface morphology, simultaneously play a critical role in defining the electrical and magnetic properties of the LSMO thin films. Therefore, while the relationship is intricate and distant, it is evident that a correlation exists between the surface morphology and the LSMO properties.

In this work, we present a machine‐learning approach to characterize ferromagnetic LSMO thin films by featurization of their surface morphology. By employing an ensemble learning method, we directly model the non‐linear correlations between the surface morphology of LSMO thin films and their physical properties. We systematically investigated the electronic and magnetic properties of LSMO thin films grown at different synthesis conditions. Focusing on the metallicity, magnetization, and ferromagnetic transition properties, the LSMO thin films could be categorized into five representative types. Importantly, we demonstrate that each type of LSMO thin film exhibits a unique surface morphology, enabling accurate classification of the films through our machine learning techniques. This result reveals that the surface morphology encompasses hidden information regarding the electronic and magnetic properties of LSMO thin films. Additionally, since the surface morphology of ferromagnetic oxide thin films is closely related to the microscopic structural distortions and symmetry, these results open new avenues for investigating the microscopic structural features and corresponding magnetic characteristics of ferromagnetic oxide thin films through surface morphology analysis.

## 2 Intricate Correlation Between Surface Morphology and Material Properties

**Figure 1:**
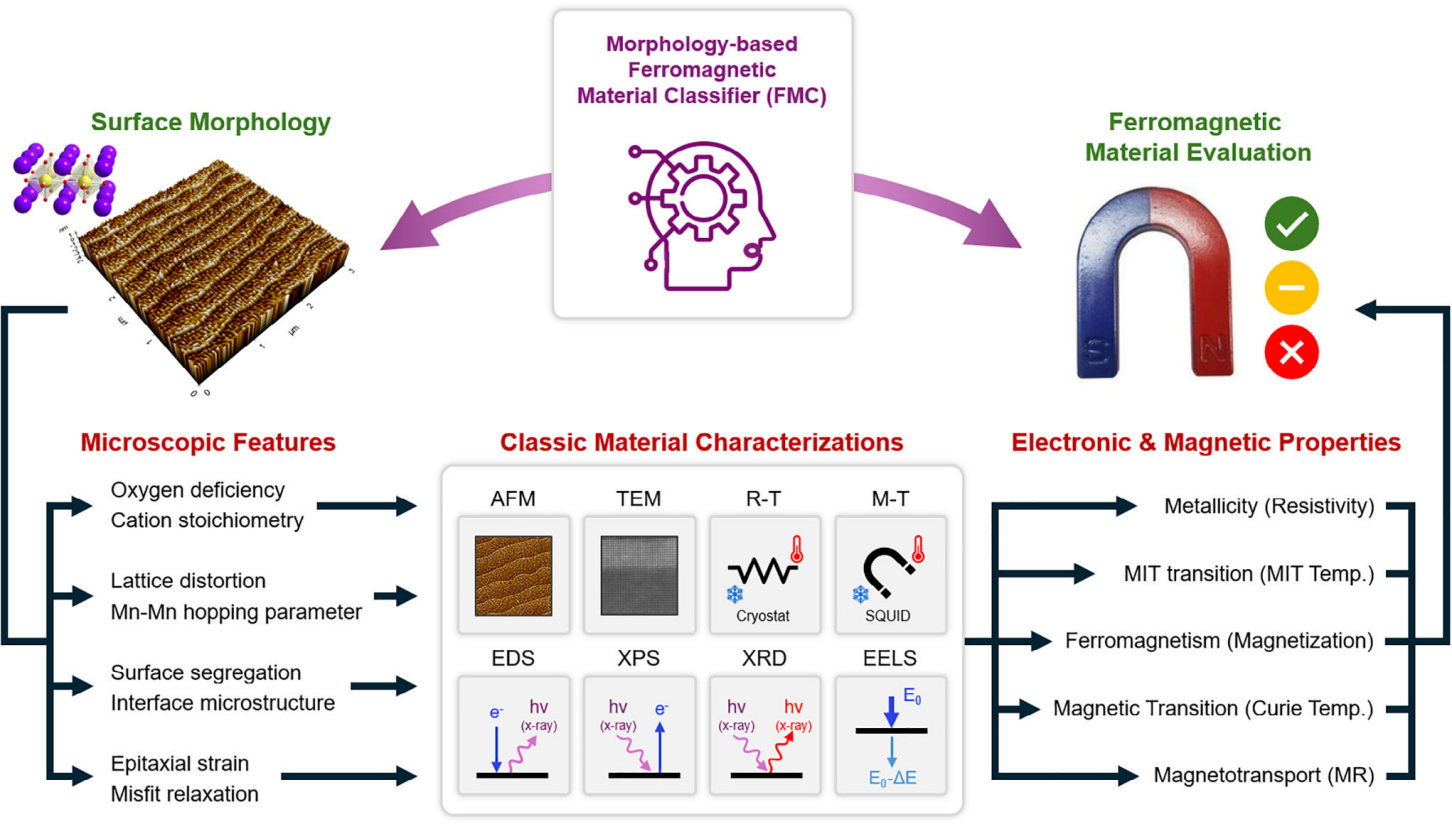
Complex correlation between the surface morphology of LSMO thin films and their electronic and magnetic properties. The lower part of this diagram shows the classical material characterization process for understanding the electronic and magnetic properties of ferromagnetic materials based on microscopic features. The upper part demonstrates how machine learning methods can model complex and non‐linear correlations between surface morphology and material properties.

**Figure** **1** shows the schematic illustration of how the surface morphology of LSMO thin films can be correlated with their electronic and magnetic properties. Since the surface morphology and electronic, magnetic properties are expected to be intricately correlated through numerous interdependencies, we first consider which specific material features predominantly establish this correlation (see the bottom left panel in Figure 1). The stoichiometry of LSMO is one of the important features that directly influence both the surface morphology and the film properties. The oxygen deficiency and the cation off‐stoichiometry are known to cause surface segregation or interface microstructures.^[^ ^25^, ^26^ ^]^ Although the direct relationship between the surface structure and the electric/magnetic properties has not been clarified, the effect of off‐stoichiometry on the material properties has been extensively studied.^[^ ^27^ ^]^ Another important feature is the epitaxial strain arising from the lattice mismatch between the LSMO and its substrate. The strain‐induced structure distortion and its relaxation are closely related to the Mn–Mn hopping parameter, significantly influencing the electronic structure and magnetic ordering in the LSMO.^[^ ^28^ ^]^ Previous studies have focused on precisely controlling these material features (e.g., stoichiometry and strain) and evaluating the corresponding changes in microscopic structures by various characterization techniques (the bottom middle panel in Figure 1),^[^ ^29^, ^30^, ^31^, ^32^, ^33^ ^]^ seeking to establish a definitive correlation between them. The microscopic structures, such as octahedral distortion and Mn‐O‐Mn buckling angle, have provided important clues to understanding the characteristic properties of LSMO thin films, such as metallicity, magnetization, and ferromagnetic transition behaviors (the bottom right panel in Figure 1).

This whole process of classic material characterizations enabled a deep understanding of LSMO. However, due to the highly complex correlations, it is still challenging to directly estimate how a small perturbation affects the LSMO properties. Therefore, recognizing that all of these material features and microscopic structures are reflected in surface morphology, we aim to establish a direct correlation between the surface morphology and the electronic, magnetic properties of LSMO thin films (the upper panel in Figure 1). By developing the socalled morphology‐based ferromagnetic material classifier (FMC), that is based on an ensemble machine learning method,^[^ ^34^ ^]^ we demonstrate that the surface morphology can represent the underlying physical properties of LSMO.

## 3 Electronic and Magnetic Characterizations of LSMO Thin Films

**Figure 2:**
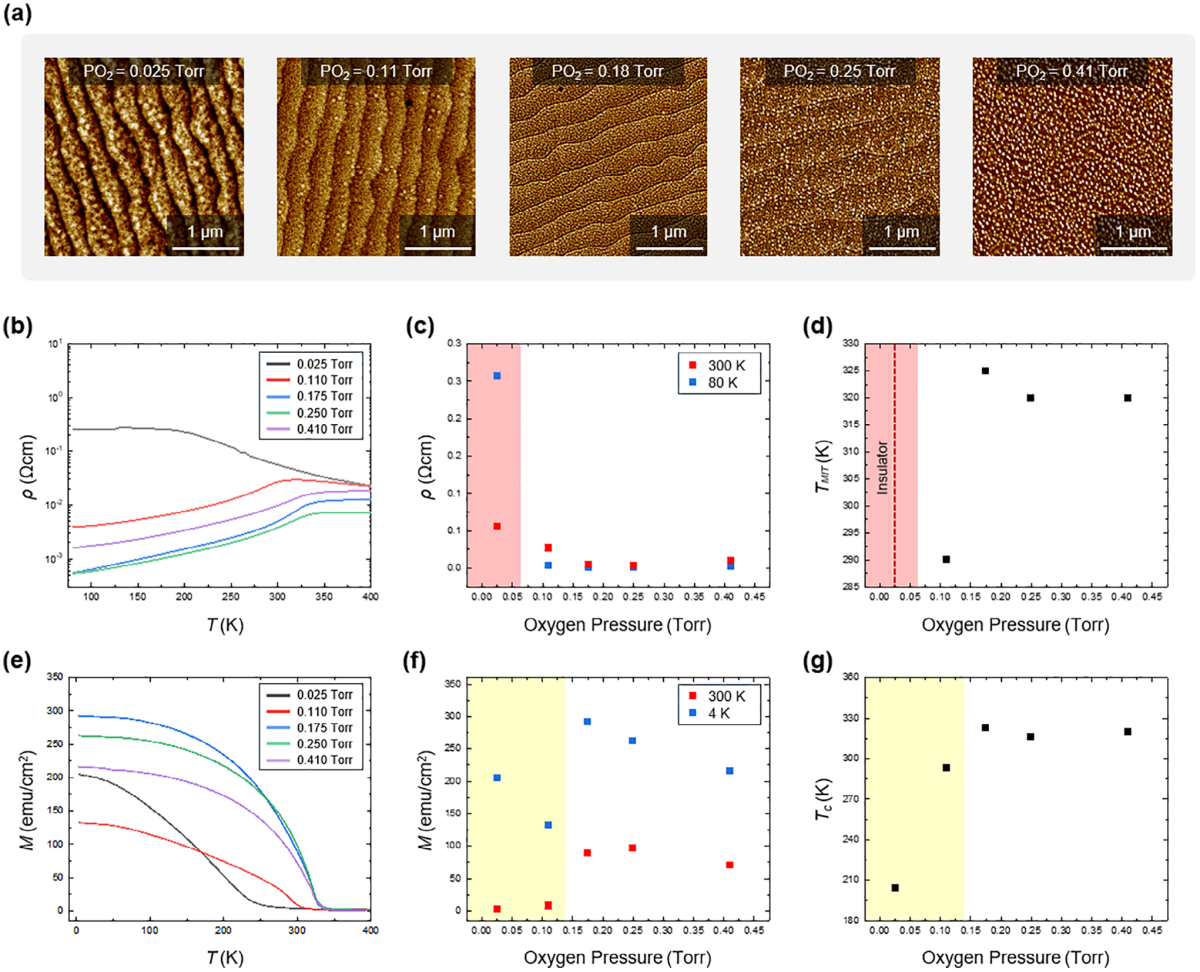
Surface morphologies and electric/magnetic properties of LSMO thin films. a) Atomic force microscopy (AFM) images were obtained from representative five types of LSMO thin films. The oxygen partial pressure (PO_2_) used for growing each thin film is displayed at the top of the images. b) Temperature dependence of the electrical resistivity *ρ* of the five types of LSMO thin films. Note that only the film grown at PO_2_ of 0.025 Torr shows insulating behavior. c) The *ρ* values were measured at 300 K (red) and 80 K (blue). d) *T_MIT_* values obtained from the same samples. The data in red‐colored regions in (c) and (d) show the characteristics of the insulating LSMO sample. In the case of the insulating LSMO sample, *T_MIT_* is not defined. e) Magnetization versus temperature (*M–T*) curves measured from the same samples. f) *M* values measured at 300 K (red) and 4 K (blue). g) *T_c_* values obtained from the same samples. The data in yellow‐colored regions in (f) and (g) exhibit inferior magnetic properties probably due to the Sr off‐stoichiometry.

To investigate the surface morphology, we prepared five types of LSMO thin films synthesized under different growth conditions. The detailed procedure for film growth is described in the Experimental Section. In brief, the LSMO thin films were epitaxially grown on SrTiO_3_ (STO) (001) substrates by pulsed laser deposition (PLD). To systematically control the surface structure and corresponding physical properties, we grew each LSMO thin film by varying only the oxygen partial pressure (PO_2_), while keeping all other conditions fixed. The PO_2_ was controlled to be 0.025, 0.11, 0.18, 0.25, and 0.41 Torr for each film. At higher PO_2_, the crystalline quality of LSMO degrades significantly, so we did not use a PO_2_ higher than 0.41 Torr. Although the growth temperature is another crucial parameter in determining the properties of LSMO, we did not use it as a control parameter. This is because the growth temperature collectively influences various factors during the material synthesis process, such as the substrate's surface energy and adatom diffusivity. Additionally, it is associated with secondary effects, such as defect migration and surface reconstruction, making it difficult to directly correlate the growth temperature with the resulting LSMO properties. To avoid the thickness‐dependent strain relaxation effects, the thickness of all the LSMO films was kept as 40 nm. The surface morphology of the as‐grown LSMO thin films was measured by atomic force microscopy (AFM) (**Figure** **2**a). It is clearly seen that the surface structures of the LSMO thin films differ from one another, each exhibiting a unique morphology. Unlike the surface morphology, which is highly sensitive to PO_2_, the crystalline structure of these LSMO thin films showed no significant difference, as confirmed by our XRD analysis (Figure S1, Supporting Information).

The AFM images show that the roughness *R* of the films increased as the PO_2_ increased (i.e., the concentration of oxygen vacancy decreased) (Figure S2, Supporting Information). The *R* is defined as the root mean square average of profile height deviations from a mean line, which can be written as $R=\sqrt{\frac{1}{L}\int_{0}^{L}z{\left(x\right)}^{2}dx}$, where *L*, *x*, and *z* represent the sampling length, scanning direction (in‐plane), and height deviation (out‐of‐plane), respectively. The LSMO thin film grown at 0.025 Torr exhibited the smallest *R* of ≈0.367 nm, while the film grown at 0.41 Torr showed the largest *R* of ≈2.24 nm. A granular surface structure appeared in the LSMO thin film grown at 0.18 Torr. As the PO_2_ was increased up to 0.41 Torr, the film exhibited significantly larger granules, resembling metal particles precipitated on the surface. It has been reported that the Sr surface segregation occurs in LSMO films as the PO_2_ is significantly low.^[^ ^19^ ^]^ However, given that the surface particles became larger with increased PO_2_, it is unlikely that Sr segregation occurred. It is more likely that the surface segregation at higher PO_2_ is related to Mn ions.^[^ ^20^, ^35^ ^]^ The slight off‐stoichiometry of Mn results in its precipitation on the surface, changing the surface morphology as well as Mn valence. In particular, the ratio between Mn^3+^ and Mn^4+^ is known to mainly determine the electrical and magnetic properties of LSMO. It has been also reported that not only the typical Mn^3+^ but also Mn^2+^ ions are present on the surface of oxygen‐deficient LSMO films, which leads to a rough surface morphology.^[^ ^36^ ^]^ These Mn‐related non‐ideal surface structures, including all the epitaxial islands, precipitates, and defects, alter the electronic structure of LSMO by increasing the occupation of Mn3*d e_g_* states. This implies that these five types of LSMO thin films, which have shown different surface structures, are expected to have distinct electronic and magnetic properties.

Figure 2b shows the temperature *T* dependence of the electrical resistivity *ρ* of the five types of LSMO thin films. The LSMO thin film grown at PO_2_ lower than 0.025 Torr exhibited an insulating behavior, whereas all the other films grown at higher PO_2_ showed metallic characteristics. The *ρ* measured at room temperature (300 K) and 80 K are given in Figure 2c. The LSMO films grown at PO_2_ ranged from 0.11 to 0.41 Torr and exhibited quite low *ρ* values, with the film grown at 0.25 Torr having the lowest *ρ* of ≈0.0038 Ω∙cm. Figure 2d shows the critical temperature (*T_MIT_*) of metal‐to‐insulator transition (MIT) for each sample. We define the *T_MIT_* as the temperature at which the first derivative of *ρ‐T* curve reaches its maximum (Figure S3, Supporting Information). The LSMO thin film grown at PO_2_ of 0.175 Torr showed the highest *T_MIT_* of 325 K. Based on these electronic properties, it is evident that the optimal metallicity can be obtained by growing LSMO films at PO_2_ ranging from 0.175 to 0.25 Torr.

In addition, we investigated the impact of PO_2_ on the magnetic ground state of LSMO. Figure 2e shows the magnetization versus temperature (*M–T*) curves measured from the same LSMO thin films. The *M* values at room temperature and 4 K are presented in Figure 2f. At room temperature, we find that the magnitude of *M* directly reflects the metallicity of the LSMO thin film. The films grown at PO_2_ of 0.175 and 0.25 Torr, which exhibited the lowest *ρ*, showed the highest *M* values at room temperature. At 4 K, the film was grown at PO_2_ of 0.175 Torr and exhibited the maximal *M* of 292 emu cm^−2^. The Curie temperatures (*T_c_*) of the LSMO thin films, defined as the peak temperature in differential *M–T* curves *dM*/*dT*, are given in Figure 2g (Figure S4, Supporting Information). The films grown at PO_2_ of 0.025 and 0.11 Torr exhibited *T_c_* lower than 300 K. In contrast, all the films grown at PO_2_ higher than 0.175 Torr showed similar *T_c_* values over 300 K. The film grown at 0.175 Torr exhibited the maximal *T_c_* of 323 K. Note that the magnetic properties of LSMO is mainly determined by the exchange interactions between the Mn ion spins.^[^ ^18^ ^]^ In the optimal LSMO film (grown at PO_2_ of 0.175 Torr), the Mn^3+^‐O‐Mn^4+^ double exchange interaction is expected to be significantly enhanced. This interaction is influenced by several factors, such as oxygen stoichiometry, A‐site (i.e., La/Sr) disorder, and strain from lattice mismatch with the substrate. In the optimal LSMO film, these factors collectively maintain Mn‐O bond angles close to 180°, maximizing electron hopping and strengthening ferromagnetic coupling.^[^ ^37^, ^38^ ^]^ As a result, this LSMO film exhibits excellent magnetic properties with a high *T_c_*.

Through these characterizations, we confirmed that the five types of LSMO thin films, grown at different PO_2_, exhibit distinct surface morphologies along with unique electronic and magnetic properties. Now we aim to establish a definite correlation between the surface morphology and the measured material properties by employing a machine learning approach.

## 4 Morphology‐based Ferromagnetic Material Classifier (FMC)

**Figure 3:**
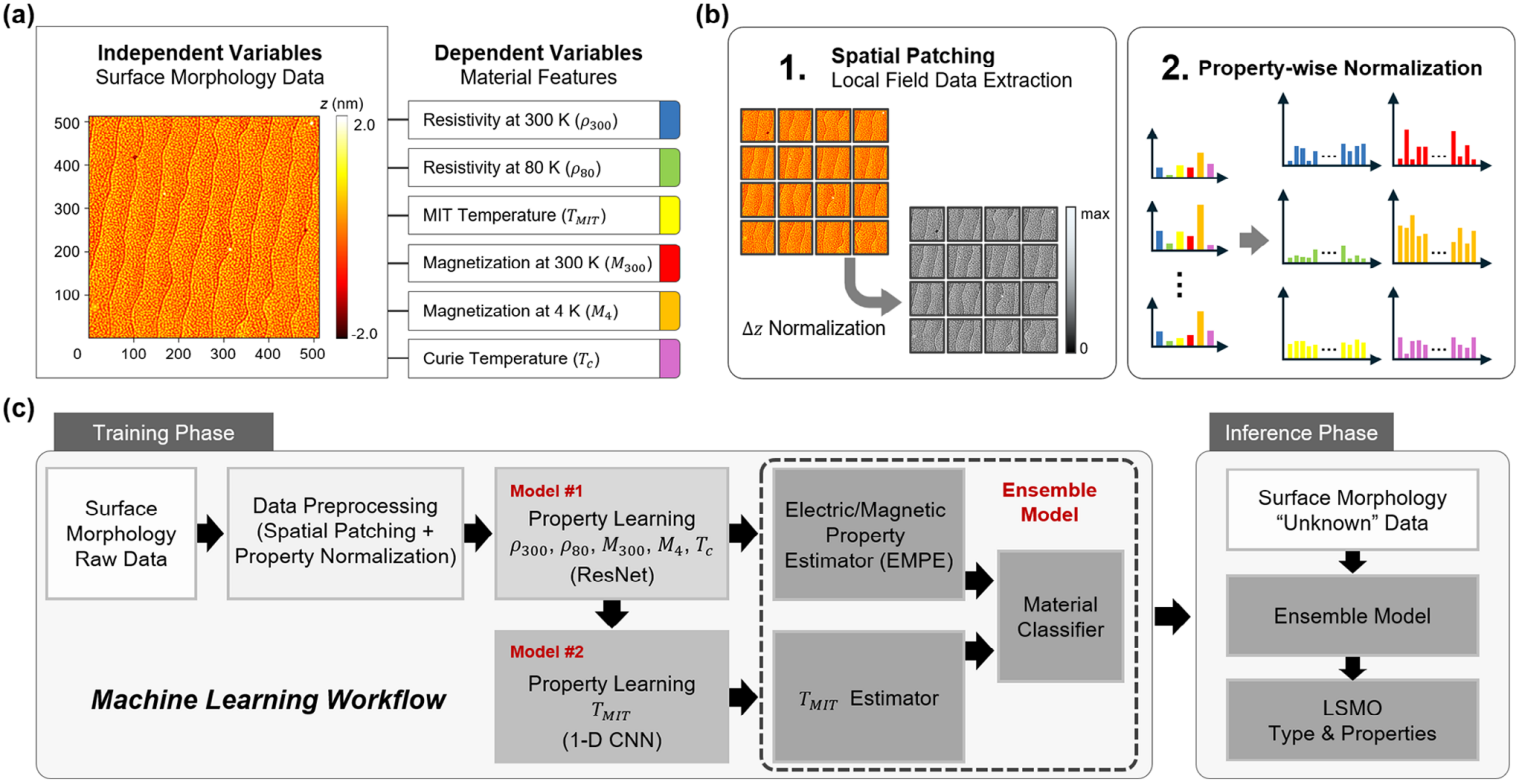
Ensemble machine learning‐based ferromagnetic material classifier (FMC). a) Machine learning problem formulation with the morphology data (AFM images) and a set of material properties. b) Data pre‐processing: (1) Spatial patching for local field data extraction and (2) Property‐wise normalization. c) The holistic view of the machine learning workflow. Our ensemble model consists of three separate artificial neural networks, one for the electric/magnetic property estimator (EMPE) for *ρ_300_*, *ρ_80_*, *M_300_*, *M_4_*, and *T_c_*, another for the *T_MIT_* estimator, and the other for lastly the material classifier for distinguishing LSMO types. Once trained, this ensemble model can estimate feature parameters directly from AFM images of LSMO thin films and classify them into one of the five major types.

We analyze inherent data patterns in morphology data and estimate the corresponding material properties using the morphology‐based FMC, that is based on an ensemble machine learning method. Given its effectiveness in integrating knowledge from multiple models,^[^ ^39^, ^40^ ^]^ ensemble learning is considered as an optimal approach for developing the morphology‐based FMC (Figure S5, Supporting Information). **Figure** **3**a shows how to design a machine learning problem with the morphology data and a set of material properties. We consider the surface morphology of LSMO thin films as independent variables and their material properties as dependent variables. To comprehensively characterize LSMO thin films, we define six material property parameters as dependent variables: 1) *ρ* at 300 K (*ρ_300_*), 2) *ρ* at 80 K (*ρ_80_*), 3) *T_MIT,_* 4) *M* at 300 K (*M_300_*), 5) *M* at 4 K (*M_4_*), and 6) *T_c_*. Then, the goal of the machine learning model is to accurately estimate these dependent variables when unknown morphology data is provided.

We prepared 500 AFM images of 512 × 512 pixels for model training (Figure S6, Supporting Information). These AFM images were obtained from a total of 500 measurements, with 100 images collected for each of the five types of samples grown under different PO_2_ conditions. For efficient training, the morphology raw data were pre‐processed as described by Figure 3b. First, we extract 16 image patches from each original morphology data such that each dimension is divided into 4 subsets. This patch size of 128 × 128 pixels enables each extracted patch to contain at least two step‐and‐terraces. Then, to avoid errors arising in the data processing stage, we rescale the pixel values so that the minimum value becomes 0. We did not scale down the maximum pixel value because it resulted in reduced accuracy of the output models (Figure S7, Supporting Information). Second, the six material property values are normalized such that the values lie between 0 and 1 using the min‐max normalization method. These data pre‐processing steps make the raw data suitable for training machine learning models by mitigating the negative effects of outliers.

Including the pre‐processing steps, the overall workflow of the morphology‐based FMC is given in Figure 3c. Note that the insulating LSMO thin films grown at the lowest PO_2_ of 0.025 Torr do not show MIT behavior. Therefore, we design two separate artificial neural networks, Model #1 for estimating the electrical and magnetic properties (i.e., ρ_300_, ρ_80_, *M*_300_, *M*_4_, and *T_c_*) and Model #2 for estimating *T_MIT_*. Once model #1 is well‐trained on the whole data, we can first differentiate the morphology of insulating LSMO films from those of metallic films using their estimated properties. Subsequently, we train model #2 exclusively on data from metallic films to enable it to accurately estimate *T_MIT_*. Finally, by stacking a small additional model upon these two independently‐trained models, we build up an ensemble model that classifies the morphology data into one of five representative LSMO types, which will be discussed in the following. More detailed descriptions of each model are provided in Figure S8 (Supporting Information).

**Figure 4:**
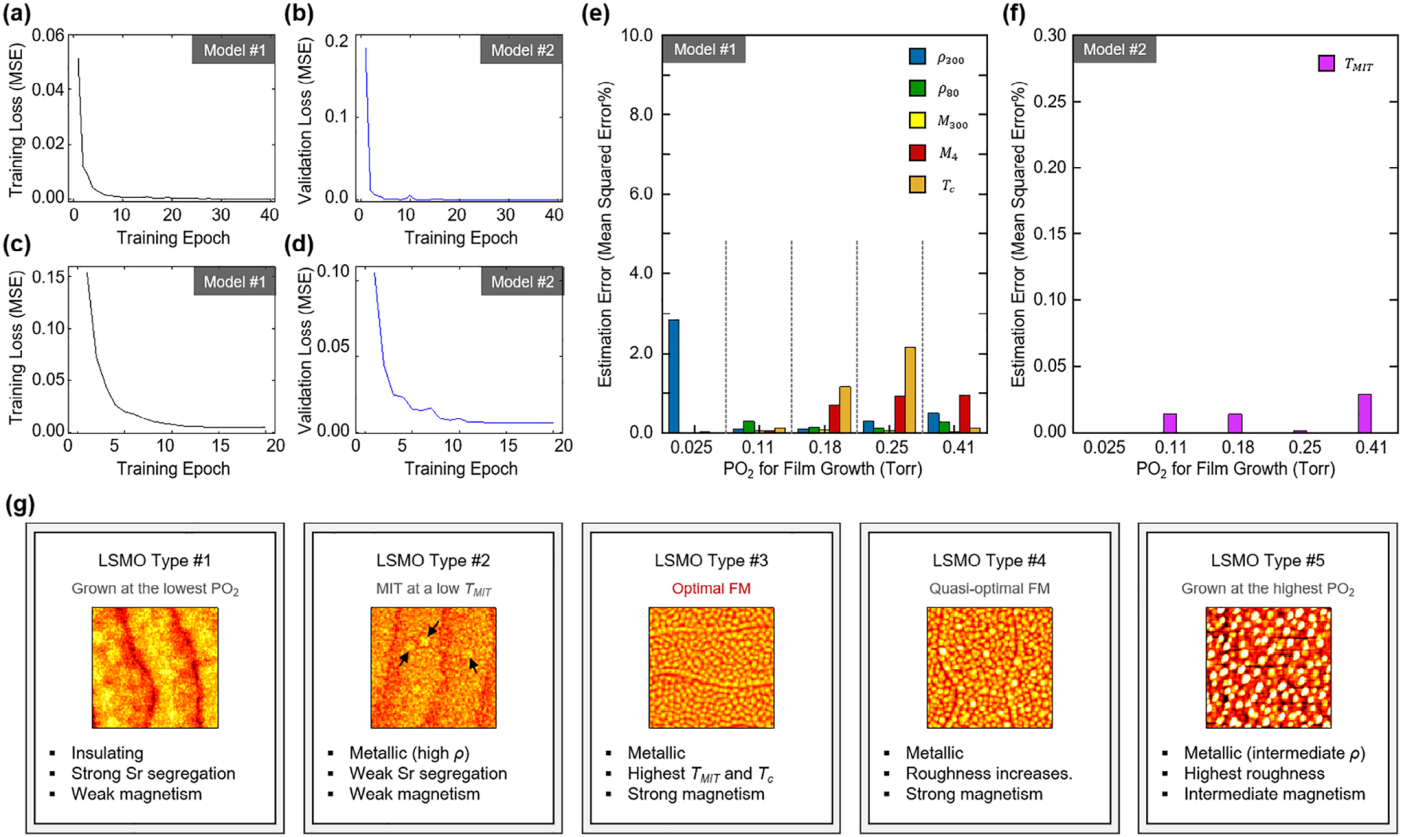
Five representative types of LSMO thin films classified by ensemble FMC model. a,b) Training loss curves of (a) model #1 and (b) model #2, respectively. c,d) The corresponding validation loss curves for (c) model #1 and (d) model #2. The loss function is mean squared error (MSE). e,f) Estimation errors of the five electric/magnetic properties and *T_MIT_*, estimated from unknown morphology data. g) Five major types of LSMO thin films. Each box briefly describes the important properties of the LSMO thin films. The inset AFM images show the representative surface morphology of LSMO for each type. The black arrows in the AFM image for Type #2 indicate the characteristic particle‐like features on the film surface.

**Figure** **4**a,b shows the training loss curves of models #1 and #2, respectively. Figure 4c,d shows the corresponding validation loss curves. The loss function is a mean squared error (MSE). In these learning curves, the loss values converge closely to zero, indicating that the models effectively learned how to estimate material properties from the given morphology data. This result empirically proves that our models can identify inherent data patterns from the morphology data and directly map them to the corresponding material property values. Figure 4e,f show the errors of the five electric/magnetic properties and *T_MIT_* estimated from unknown morphology data, respectively. The errors are defined as $[\frac{|x_{ex}-x_{es}|}{x_{ex}}]\%$, where *x_ex_* and *x_es_* denote the expected and estimated values. Notably, all property values are accurately estimated with small error values (< 3%). Therefore, an ensemble model can be reliably built up to address the material classification problem based on these two models (Figure 3c). Finally, our ensemble model achieves 100% validation accuracy when given the validation morphology data.

This result reveals the existence of a correlation between the surface morphology and the physical parameters of LSMO thin films. Based on this correlation, we emphasize that the epitaxially‐grown LSMO thin films can be classified into five major types (Figure 4g). In brief, Type #1 represents LSMO films grown under a quite low PO_2_. These LSMO films, grown at a reducing condition, often suffer strong Sr segregation and show insulating behavior.^[^ ^19^ ^]^ Type #2 includes LSMO films grown at a slightly increased PO_2_. These films show small particle‐like features caused by Sr segregation on the surface (see black arrows in Figure 4g). While they exhibit ferromagnetic metallic behavior, their *M* values are low, and their *ρ* are relatively high. Type #3 represents an optimal ferromagnetic LSMO films. These films are expected to be stoichiometric and exhibit characteristic surface features with a relatively small roughness. They clearly show the high *M* and the low *ρ* values. The highest *T_MIT_* and *T_c_* are another important features of the optimal LSMO films. The Type #4 LSMO films exhibit similarly strong ferromagnetic properties to those in Type #3. However, they show slightly lower *M* and *T_MIT_* values, along with a noticeable increase in surface roughness. This is likely due to the Mn off‐stoichiometry.^[^ ^17^ ^]^ Lastly, Type #5 includes LSMO films grown at a high PO_2_. These LSMO films show extremely high surface roughness and the intermediate *M* and *ρ* values, indicating that the PO_2_ is not suitable for growing the ferromagnetic LSMO. These analyses imply that the oxygen environment directly influences the cation stoichiometry and the defect formation energy in LSMO thin films, determining their surface structures and ferromagnetic properties. Since various synthesis conditions, such as laser fluence, temperature, and deposition rate, collectively influence the structure and composition of LSMO, the numerical values of PO_2_ may not serve as direct indicators. However, based on our analyses, we suggest that PO_2_ can be an important relative indicator for optimal LSMO growth. Lastly, our study confirms that the surface morphology of the as‐grown LSMO films provides accurate and fruitful information regarding their electronic and magnetic properties.

## 5 Conclusion

In summary, by employing an ensemble machine learning method, we successfully modeled the non‐linear correlations between the surface morphology of LSMO thin films and their representative physical properties, including the resistivity, MIT temperature, Magnetization, and Curie temperature. Our FMC machine learning models could accurately estimate the material property parameters from the surface morphology image obtained by AFM, enabling the categorization of LSMO thin films. Given the high accuracy and efficiency of our FMC ensemble model, these results open up opportunities to better understand the intricate relationship between the unstable Mn ionization state, the microscopic change in Mn‐O‐Mn structures, and the corresponding LSMO properties. Additionally, we discuss the applicability of our FMC approach to other ferromagnetic materials. Since FMC is designed to identify inherent correlations between surface morphology patterns and material properties, it can, in principle, effectively analyze any material system that exhibits such correlations. However, if there exists a material whose surface morphology does not sensitively reflect material parameters, such as stoichiometry and microscopic lattice distortions, that influence its intrinsic electric and magnetic properties, analyzing it using our FMC approach would be challenging. Nevertheless, in most ferromagnetic materials, surface morphology is correlated with various material parameters. Therefore, we emphasize that this machine learning approach offers a promising way to explore a wide range of strongly correlated ferromagnetic systems by featurization of surface structures or other readily‐available large datasets.

## 6 Experimental Section

### Preparation of LSMO/STO Heterostructures

The single‐crystalline LSMO thin films were grown on TiO_2_‐terminated STO (001) substrates by PLD. As‐received STO substrates (Shinkosha Co., Ltd.) were first etched by buffered‐HF for 80 s and then thermally annealed at 900 °C for 6 h. Subsequently, a stoichiometric LSMO target was ablated by an excimer laser (λ = 248 nm) with a repetition rate and fluence of 3 Hz and 1.3 J cm^−2^, respectively. During the film growth, the temperature of substrates was kept as 630 °C. All the as‐grown LSMO/STO samples were cooled in an oxygen atmosphere of 1 atm to minimize the formation of oxygen vacancy defects. The LSMO deposition rate was calibrated by X‐ray reflectivity measurements.

### Surface Morphology Measurements by Atomic Force Microscopy (AFM)

The surface morphology of the samples was measured by an AFM (NX10, Park Systems). The measurements were performed using a commercial cantilever (PPP‐NCHR, Nanosensors) in noncontact mode. All the images were measured at identical conditions. The scan rate and image resolution were set to be 1 Hz and 512 × 512 pixels, respectively.

### Electrical and Magnetic Characterizations

The electrical transport characteristics of LSMO thin films were measured using a hall‐effect measurement system (HMS‐5300LTH, Ecopia). The van‐der‐pauw geometry was used for all the samples. The transport measurements were performed in a temperature range between 80 and 400 K under a magnetic field of 0.585 T and a constant current of 0.2 mA. To characterize the magnetic properties of the samples, a superconducting quantum interference device‐vibration sample magnetometer (SQUID‐VSM) measurement system (MPMS‐3, Quantum Design) was used. The LSMO samples were cooled down under an in‐plane magnetic field of 1 kOe, followed by the measurement of the *M*–*T* curve increasing temperature from 4 to 400 K.

### X‐ray Diffraction (XRD) Measurements

A SmartLab (Rigaku) high‐resolution X‐ray diffractometer with λ = 1.54184 Å was used for the XRD measurements. Theta‐2theta scans were carried out around the STO (002) reflection for LSMO/STO heterostructures.

## Conflict of Interest

The authors declare no conflict of interest.

## Supporting information



Supporting Information

## Data Availability

The data that support the findings of this study are available from the corresponding author upon reasonable request.;
