# Supplementary material for: Machine Learning Approach to Characterize Ferromagnetic La0.7Sr0.3MnO3 Thin Films via Featurization of Surface Morphology
Source: Adv Sci (Weinh). 2025 Apr 26;12(23):2417811. doi: 10.1002/advs.202417811 (PMC12199370; doi:10.1002/advs.202417811)
Supplement: Supplementary file 1 — Supporting Information [file ADVS-12-2417811-s001.docx]

Supporting Information

Machine Learning Approach to Characterize Ferromagnetic La_1-x_Sr_x_MnO_3_ Thin Films via Featurization of Surface Morphology

Sanghyeok Ryou^†^, Jihyun Lim^†^, Minwoo Jang, Kitae Eom, Sunwoo Lee*, and Hyungwoo Lee*


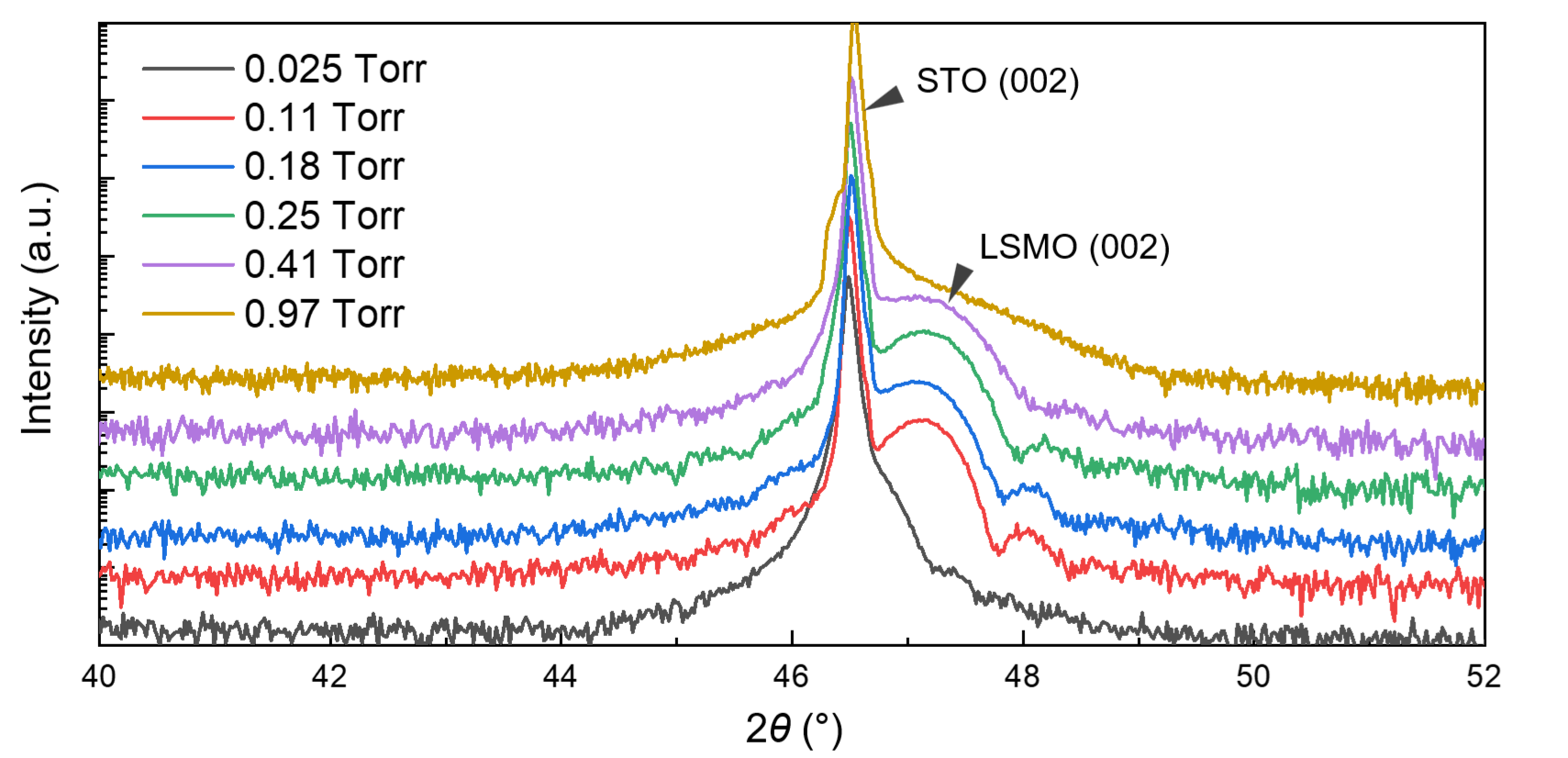


**Figure S1.** X-ray diffraction (XRD) theta-2theta scan of the La_0.7_Sr_0.3_MnO_3_/SrTiO_3_ (LSMO/STO) heterostructures grown at different oxygen partial pressures (PO_2_). XRD spectra of LSMO films grown at PO_2_ ranged from 0.025 to 0.970 Torr. Note that at PO_2_ below 0.41 Torr, all the LSMO films were epitaxially well grown on STO (001) substrates with a single out-of-plane orientation. The LSMO (002) film peaks are consistently separated from the substrate peak (i.e., STO (002)) to a higher 2theta angle, indicating that the c-axis lattice constant of the LSMO films is smaller than that of the STO substrate. We find that the c-axis lattice constant of LSMO thin films decreases with increasing PO_2_. This slight shift of LSMO (002) peak is associated with the formation of oxygen vacancies during film growth. Charged oxygen vacancies are known to increase the unitcell volume of the perovskite crystal lattice. However, as shown by these XRD spectra, it is evident that the formation of oxygen vacancies does not significantly affect the crystalline quality of our LSMO films. On the other hand, LSMO films grown at PO₂ higher than 0.41 Torr, such as 0.97 Torr (dark yellow line), exhibited severely degraded crystalline quality. This result is well consistent with previous studies.[41,42] Thus, we set the PO_2_ of 0.41 Torr as the upper limit of growth window.

**
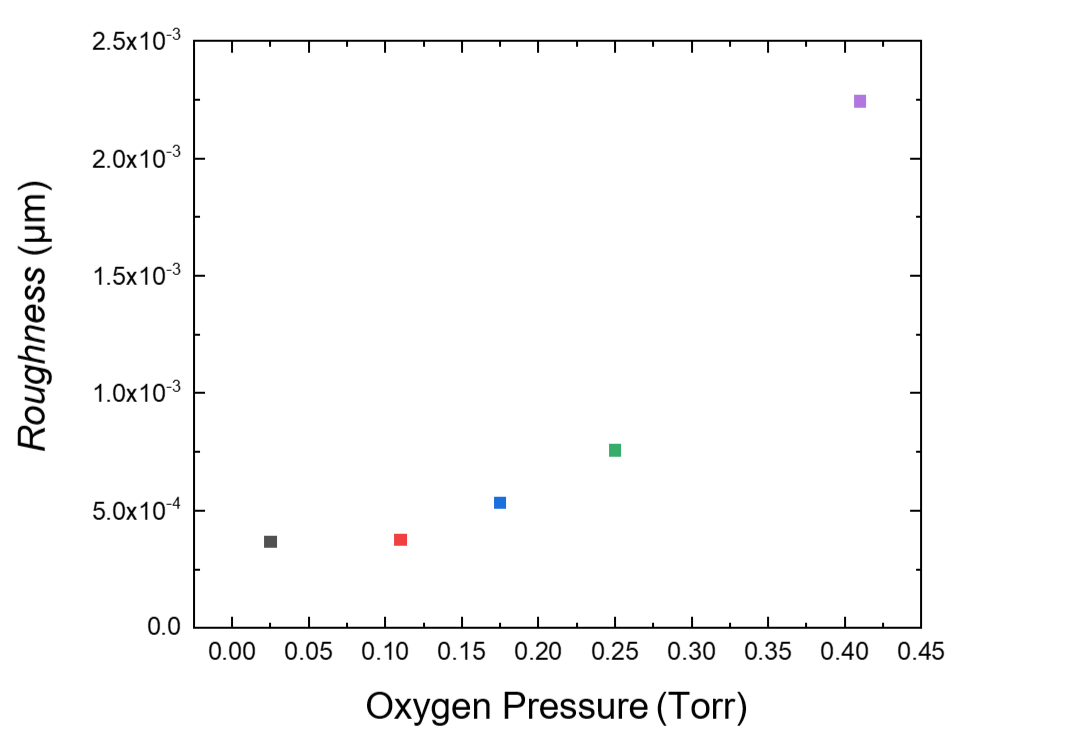
**

**Figure S2.** Roughness of LSMO thin films grown at different PO_2_. In this work, we define the roughness *R* as root mean square average of profile height deviations from a mean line, which can be written as $R=\sqrt{\frac{1}{L}\int_{0}^{L} {z(x)}^{2}dx}$, where *L*, *x*, and *z* represent the sampling length, scanning direction (in-plane), and height deviation, respectively. At lower PO₂ levels, the surface seggregation of Sr occurs, leading to a significant increase in roughness. However, such conditions result in significantly poor ferromagnetic properties, and therefore, we excluded such strongly off-stoichiometric regions from our study. AFM images suggest that the two LMSO thin films grown at PO₂ of 0.025 Torr and 0.11 Torr exhibit only a slight Sr seggregation with minimal roughness values. On the other hand, thin films grown at a higher PO₂ of ~0.41 Torr show a significant increase of surface roughness. In this case, the increase of the surface roughness is probably due to the Mn ions rather than Sr. Notably, these films grown at the high PO_2_ also showed degraded *M* and *ρ*. Therefore, this observation clearly indicates that there exists a correlation between the surface structure of LSMO and its electronic and magnetic properties.

**
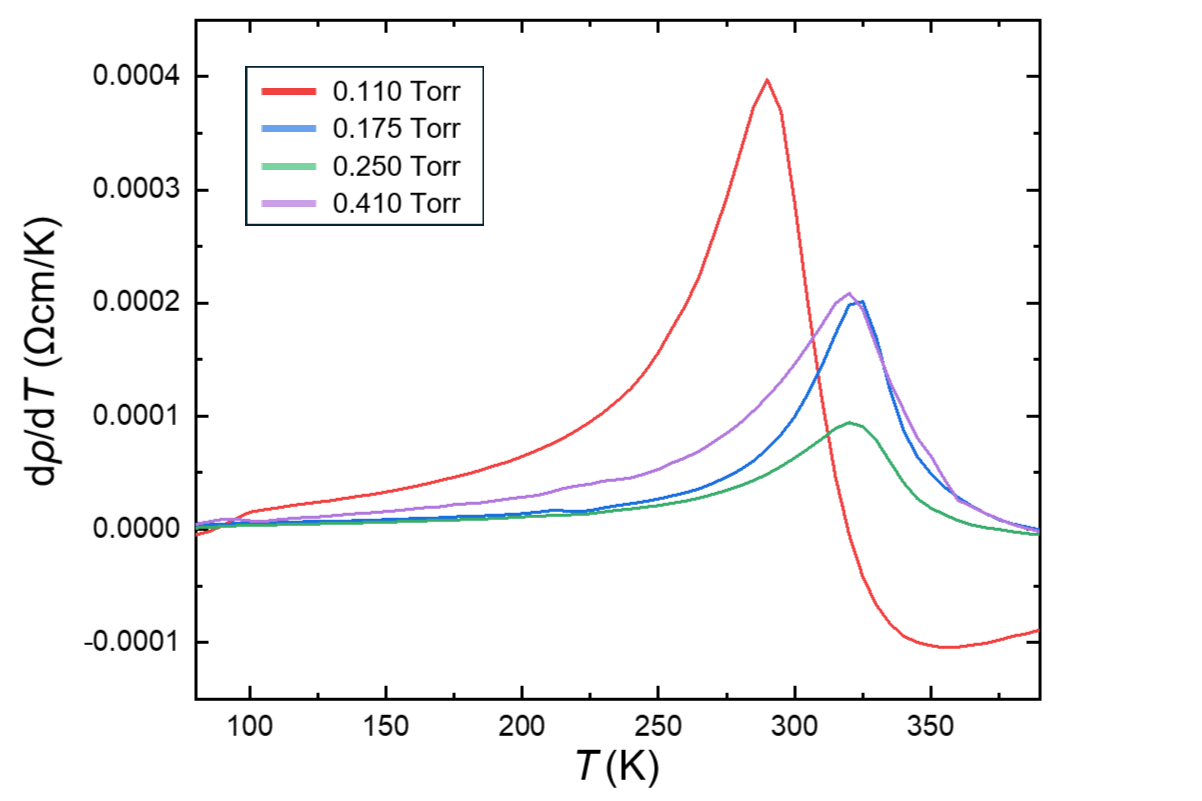
**

**Figure S3.** The first derivative of *ρ*(*T*) as a function of *T* for the LSMO thin films grown at different PO_2_. We determined the critical temperature (*T_MIT_*) of metal-to-insulator transition (MIT) for LSMO thin films using these results. We defined the *T_MIT_* as the temperature at which the first derivative of *ρ*(*T*) reaches the maximum value. The *T_MIT_* values are estimated as ~290K , ~325 K, ~320 K, ~320 K for the LSMO films grown at 0.11 Torr, 0.175 Torr, 0.25 Torr, and 0.41 Torr, respectively. As for the LSMO film grown at 0.025 Torr, the derivative plot could not be obtained because the sample was insulating.

**
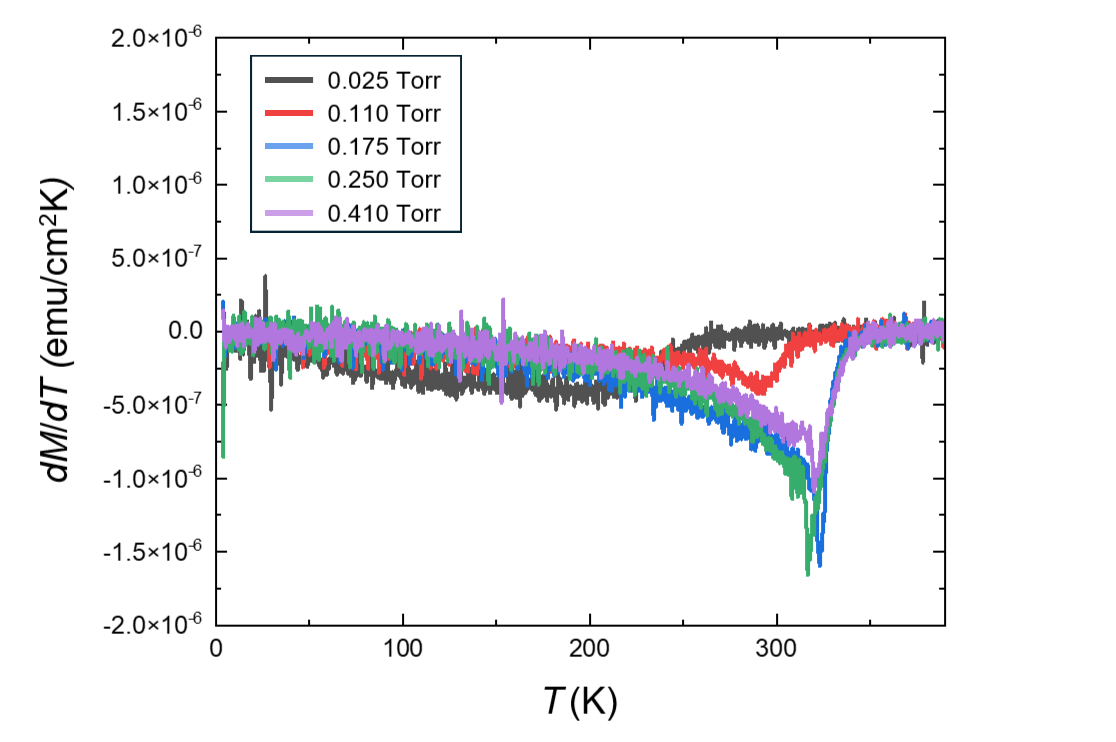
**

**Figure S4.** The first derivative of *M*(*T*) as a function of *T* for the LSMO thin films grown at different PO_2_. The Curie temperatures (*T_c_*) of the LSMO thin films were defined as the peak temperatures in these differential *M*-*T* curves *dM*/*dT*. The *T_c_* values are estimated as ~204 K , ~293 K, ~323 K, ~316 K, and ~320 K for the LSMO films grown at 0.025 Torr, 0.11 Torr, 0.175 Torr, 0.25 Torr, and 0.41 Torr, respectively. In the low PO₂ region, it is clearly seen that T_c_ increases as PO₂ increases. However, when PO₂ exceeds approximately 0.175 Torr, *T_c_* tends to converge to a constant value.

**
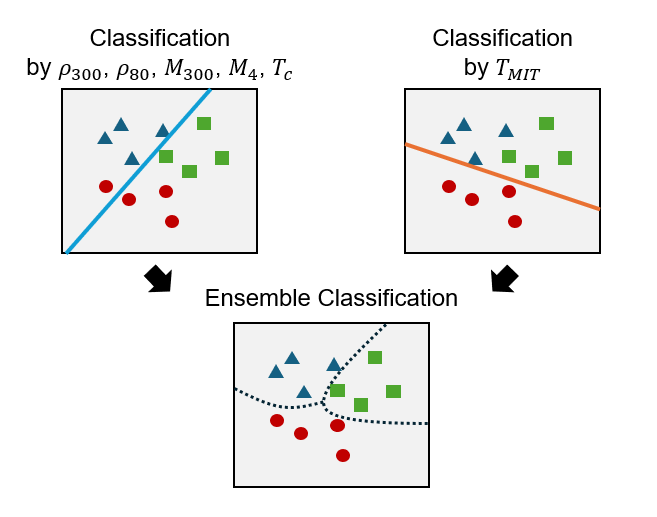
**

**Figure S5.** The ensemble learning framework built to characterize material type. One model is specified to characterize a few magnetic properties while another is designed to estimate the MIT temperature. Once these two models are well trained, we develop an extra machine learning model which learns how to differentiate one material type from another based on the two models’ material property estimations. This ensemble approach enables us to model the inherent data patterns in magnetic materials and insulators, resulting in a unified method for classifying material types.


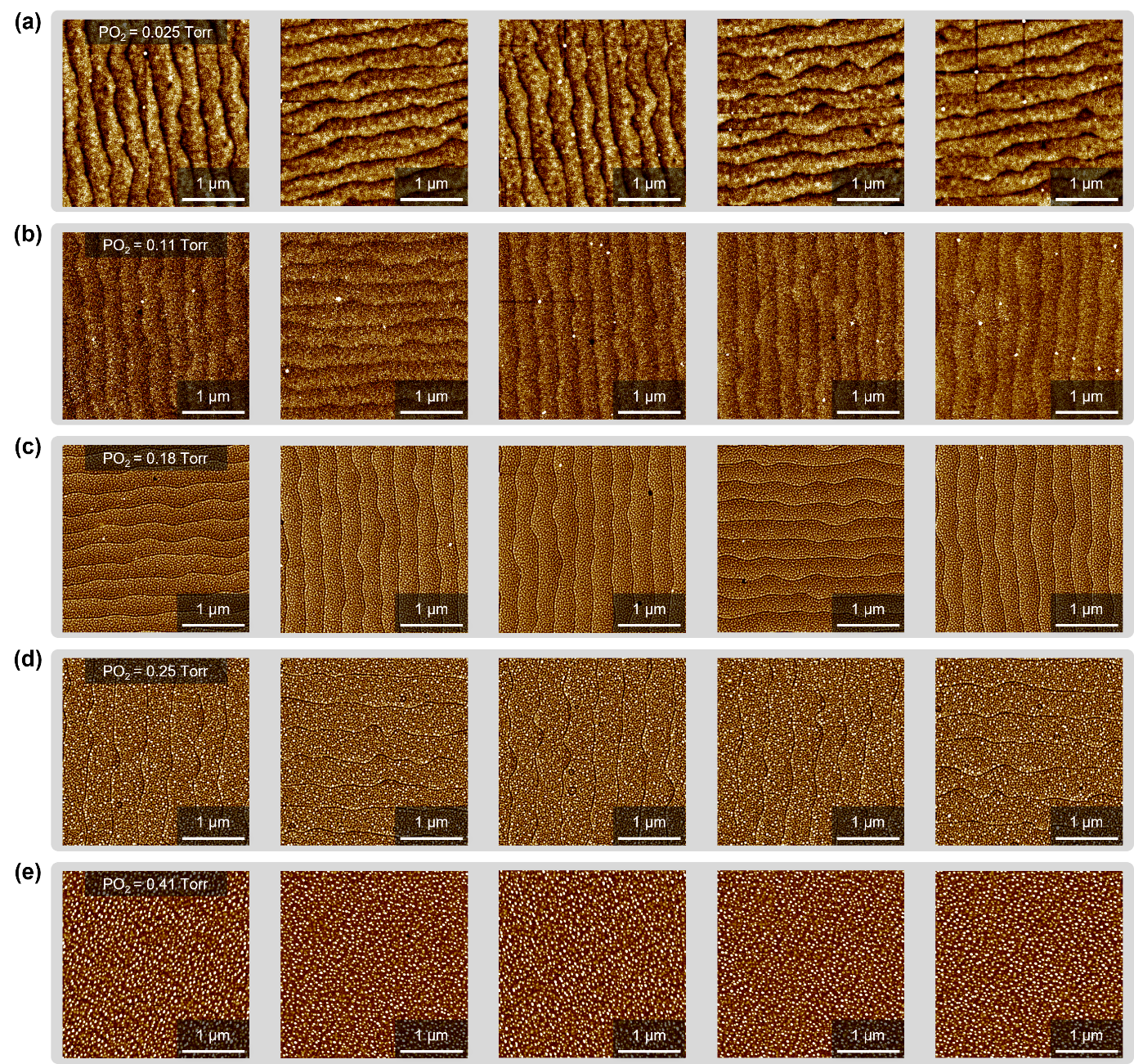


**Figure S6.** Surface morphology data for each type of LSMO thin films. These atomic force microscopy (AFM) images show the representative surface morphology of LSMO thin films grown at PO_2_ of (a) 0.025 Torr, (b) 0.11 Torr, (c) 0.18 Torr, (d) 0.25 Torr, and (e) 0.41 Torr, respectively. We measured 100 AFM images, with a resolution of 512 x 512 pixels, for each of the five types of LSMO film (i.e., grown at different PO_2_). The total 500 AFM images then were divided into 128 x 128 pixel patches, resulting in a dataset of 8,000 images. All the AFM images were obtained with the same probe tip under the same measurement condition.

**
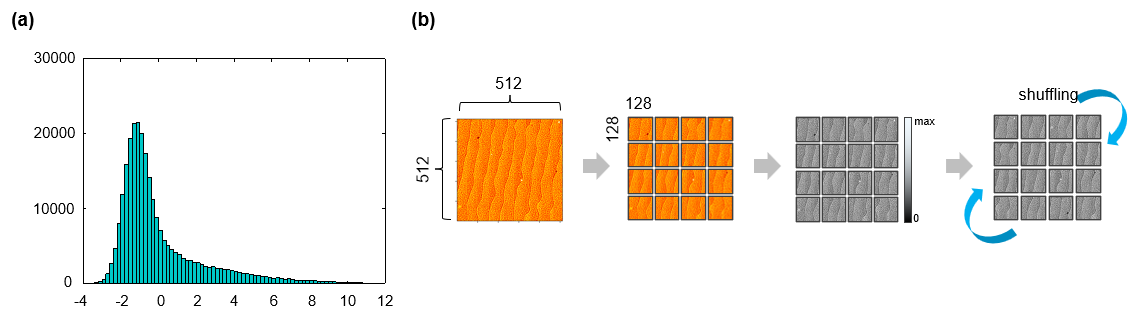
**

**Figure S7.** Morphology data preprocessing steps. Due to the flattening process of AFM images, the morphology data contain a large amount of negative values. (a) A histogram of one example morphology data sample. The first step in preprocessing is to shift the values to a positive range by adding the minimum value to all individual points. The minimum value is found in each morphology image separately. The next step is to split each image to 16 patches such that each dimension is divided into 4 subsets as shown in (b). The original data consists of 500 images each of which has $512\times512$ points. Thus, we generate 8,000 patches whose size is $128\times128$. We use 90% of those patches for training and the rest of them for validation. When training neural networks, the training patches are randomly shuffled, and a subset of them are sampled at each model update. The labels are normalized using a min-max normalization method. Each value is normalized using the following equation: $\bar{x}=\frac{x-x_{min}}{x_{max}-x_{min}}$. Nevertheless, we acknowledge that the total number of training images is relatively small. To address the potential issue of overfitting, we employed two techniques. First, we applied two data augmentation methods, spatial patching and random shuffling, to the original morphology images. These pre-processing steps introduce well-controlled noise into the data, effectively reducing overfitting. Second, we utilized a learning rate schedule specifically designed to enhance the generalization of the machine learning model. The training starts with a relatively large learning rate to accelerate convergence. After sufficient training in this high-noise regime, we gradually decrease the learning rate to help the model focus on capturing more subtle data patterns. This learning rate adjustment strategy is known to enhance generalization.[43] With these techniques, our FMC is expected to accurately predict material properties from previously unseen AFM images.

**
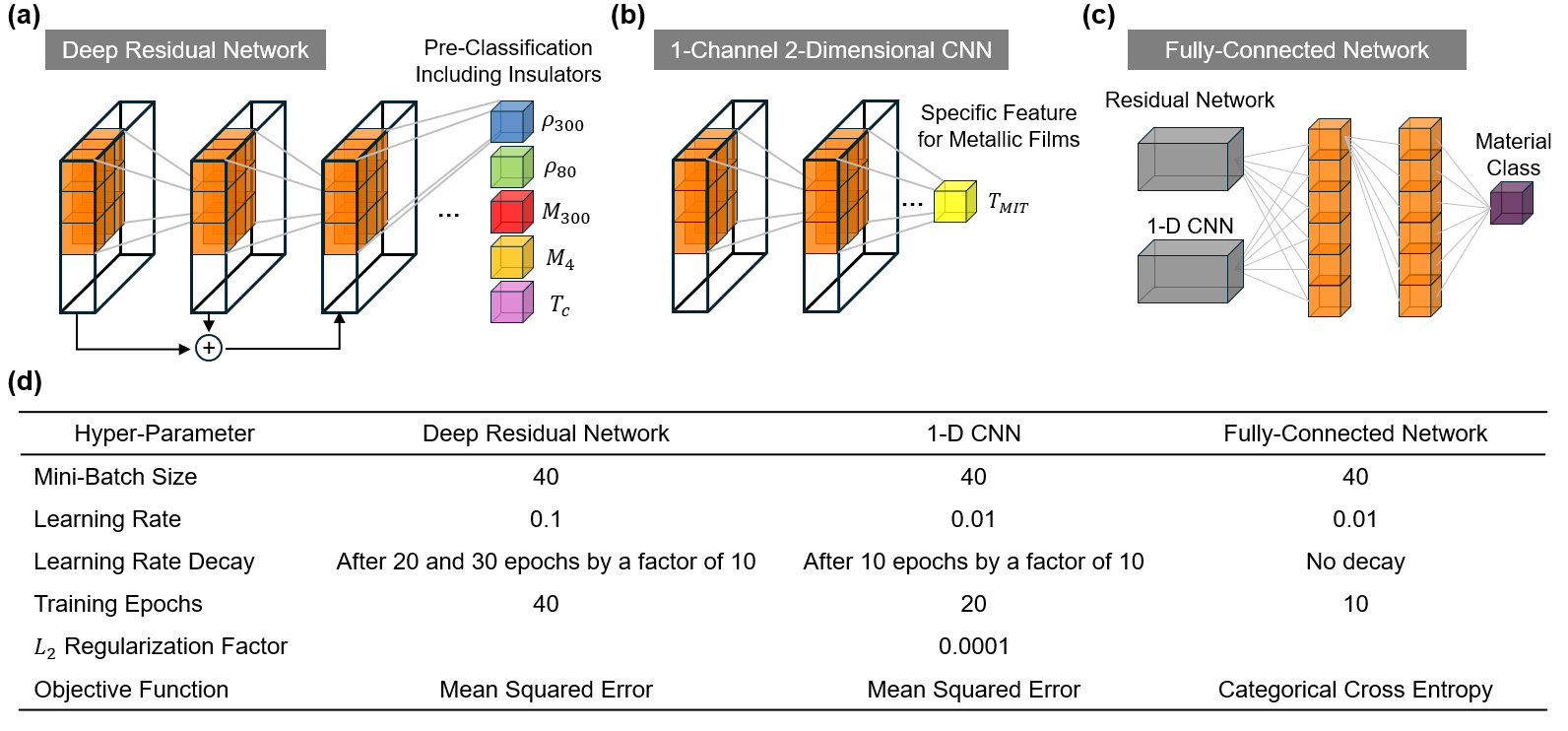
**

**Figure S8.** Hyper-parameter settings for neural network training. (a-c) Schematic illustrations for three neural networks we used in this study. The first classifier for estimating magnetic properties from morphology data is (a) a deep residual network which contains 27 layers and 276,109 parameters. The second classifier for estimating $T_{MIT}$ is (b) a convolutional neural network that has 5 layers and 657 parameters. Both models employs $L_{2}$ regularizer. The final classifier built upon these two models is (c) a fully-connected network which has 2 layers and 389 parameters. All the networks are trained using mini-batch stochastic gradient descent with momentum (momentum factor is 0.9). (d) Summarized training hyper-parameters.

**References**

[41] J. Sakai, N. Ito, and S. Imai, *Journal of Applied Physics* **2006**, 99, 8.

[42] S. Kumari, et al. *Scientific Reports* **2020**, 10, 3659.

[43] S. Lee, C. He, and S. Avestimehr, *Neural Networks* **2023**, 158, 1-14.
